# Alcohol-Based Handrubs and Influenza A

**DOI:** 10.1128/mSphere.00787-19

**Published:** 2019-11-27

**Authors:** John M. Boyce

**Affiliations:** a J.M. Boyce Consulting, LLC, Middletown, Connecticut, USA; National Institute of Allergy and Infectious Diseases

**Keywords:** influenza, hand hygiene, hand washing, prevention, respiratory infection, alcohol-based handrub

## LETTER

I read with interest the article by Hirose et al. (1), which describes the authors’ study of the relative efficacies of antiseptic hand rubbing (AHR) and antiseptic hand washing (AHW) in preventing the transmission of influenza A virus (IAV). However, the article raises several concerns.

For example, the abstract states that “Additionally, AHR and AHW effectiveness against infectious mucus adhering to the hands and fingers was evaluated in 10 volunteers.” However, the authors applied IAV to the fingertips of 10 test subjects, not to their hands, and there was no assessment of the extent to which infectious mucus adhered to the fingertips.

Several aspects of the study design raise concerns regarding the validity and generalizability of the findings reported. First, there is no description of how mucus samples (sputum samples ≥ 2 gm) were obtained. Individuals with influenza typically present with a dry, nonproductive cough (2, 3), which likely results in little, if any, visible hand contamination with sputum. Sputum production may occur with influenza (2, 4, 5), and when it does, it is not uncommon for it to result from coinfection with other respiratory pathogens such as Streptococcus pneumoniae, Haemophilus influenzae, or Staphylococcus aureus (5). However, no evidence was provided that coinfection with bacterial pathogens, which could very likely affect the viscosity of sputum, was excluded. Furthermore, sputum production is more common in elderly patients with influenza (2, 5). The text states that patients who are <20 years old were excluded, but no demographics of the individuals studied were provided. These facts raise questions about how the mucus (sputum) samples were obtained (by a sputum induction procedure performed with saline solution or by expectoration) and suggest that the individuals studied may not have been representative of the majority of individuals with influenza.

The *in vivo* studies involving the 10 test subjects were reportedly performed utilizing the American Society for Testing and Materials finger pad protocol (ASTM E1838) (6), which recommends that a tube containing the antiseptic be applied to the fingertips and be inverted 10 times during the exposure period. The article does not state that this was done, which would represent a protocol violation. Also, the protocol does not require rubbing the alcohol on the skin and, as a result, does not mimic the way that alcohol-based handrubs are used (7–9). Finally, the study utilized ethanol alone, whereas alcohol-based handrubs containing additional ingredients could conceivably yield results different from those reported by the authors.

The issues noted above raise serious questions about the generalizability and validity of the authors’ findings and their conclusion that alcohol-based handrubs have reduced effectiveness in preventing transmission of influenza virus. Because earlier studies demonstrating the effectiveness of alcohol-based handrubs used protocols that involved applying influenza virus to fingers and allowing the substance to dry in the absence of respiratory secretions (10, 11), additional *in vivo* studies of the impact of commercially available alcohol-based handrub products on influenza virus may be desirable. Also, since influenza virus is most commonly transmitted via large respiratory droplets, it should be emphasized that influenza vaccination is considered the most important means of preventing influenza (3).
